# Genomic Evidence for Bacterial Determinants Influencing Obesity Development

**DOI:** 10.3390/ijerph14040345

**Published:** 2017-03-26

**Authors:** Raphael D. Isokpehi, Shaneka S. Simmons, Matilda O. Johnson, Marinelle Payton

**Affiliations:** 1 College of Science, Engineering and Mathematics, Bethune-Cookman University, Daytona Beach, FL 32114, USA; 2 Division of Arts and Sciences, Jarvis Christian College, Hawkins, TX 75765, USA; shaneka.s.simmons@gmail.com; 3 Center of Excellence in Minority Health and Health Disparities, Jackson State University, Jackson, MS 39217, USA; marinelle.payton@jsums.edu; 4 Petrock College of Health Sciences, Bethune-Cookman University, Daytona Beach, FL 32114, USA; johnsonma@cookman.edu

**Keywords:** bacteria, *Akkermansia*, body weight, *Lactobacillus*, microbiome, obesity, overweight, probiotics, public health, universal stress proteins

## Abstract

Obesity is a major global public health problem requiring multifaceted interventional approaches including dietary interventions with probiotic bacteria. High-throughput genome sequencing of microbial communities in the mammalian gastrointestinal system continues to present diverse protein function information to understand the bacterial determinants that influence obesity development. The goal of the research reported in this article was to identify biological processes in probiotic bacteria that could influence the mechanisms for the extraction of energy from diet in the human gastrointestinal system. Our research strategy of combining bioinformatics and visual analytics methods was based on the identification of operon gene arrangements in genomes of *Lactobacillus* species and *Akkermansia* *muciniphila* that include at least a gene for a universal stress protein. The two major findings from this research study are related to *Lactobacillus plantarum* and *Akkermansia muciniphila* bacteria species which are associated with weight-loss. The first finding is that *Lactobacillus plantarum* strains have a two-gene operon that encodes a universal stress protein for stress response and the membrane translocator protein (TSPO), known to function in mitochondrial fatty acid oxidation in humans. The second finding is the presence of a three-gene operon in *Akkermansia muciniphila* that includes a gene whose human mitochondrial homolog is associated with waist-hip ratio and fat distribution. From a public health perspective, elucidation of the bacterial determinants influencing obesity will help in educating the public on optimal probiotic use for anti-obesity effects.

## 1. Introduction

Obesity is a major global public health problem requiring multifaceted interventional approaches including dietary interventions with probiotic bacteria [1,2,3,4]. High-throughput genome sequencing of microbial communities in the mammalian gastrointestinal system continues to present diverse protein function information to understand the microbial molecular mechanisms that influence obesity development [5,6]. The Gram-positive bacteria genus *Lactobacillus* includes probiotic members which have been investigated for the metabolic role in weight modification in humans [7,8].

The goal of the research reported in this article was to identify biological processes in lactobacilli that could influence the mechanisms for the extraction of energy from the diet in the human gastrointestinal system. Lactobacilli that inhabit the human gastrointestinal system or part of probiotics must respond to unfavorable conditions including stomach acid, bile acid and low oxygen. This research article contributes knowledge on environmental factors (gastrointestinal microbiota) that could influence the development of obesity [5,6], a major risk factor for diseases such as diabetes, cancer, and cardiovascular disease [9,10,11]. Proteins that contain the universal stress protein domain (Protein Family (Pfam)Accession: PF00582 or pfam00582) are known to confer diverse categories of organisms including bacteria with the ability to respond to habitat-related stress conditions. We are especially interested in universal stress proteins (USPs) because of the role of some members in the formation of the structured community of cells called biofilm. Lactobacilli biofilms have been found in the gastrointestinal tract and other body sites. For example, biofilm formation by probiotic *L. rhamnosus* GG is influenced by the presence of bile and mucins, acidic conditions and high osmolarity [12]. Since species in *Lactobacillus* have diverse habitats and roles, we focus our current evaluation on lactobacilli that have been investigated for a metabolic role in body weight modification in humans. Weight protection lactobacilli strains are found in *L. plantarum* and *L. gasseri*, while weight gain lactobacilli strains are found in *L. reuteri*, *L. acidophilus*, *L. fermentum*, *L. sakei* and *L. ingluviei* [8].

Since bacteria genes can be organized to optimize the steps in biological processes [13], the analysis of the gene neighborhood of genes for universal stress proteins could provide clues to their habitat-related functions. As part of the prediction of gene function, several bioinformatics resources provide predictions of the gene neighborhood of bacterial genes including the presence of operons (groups of genes that are transcribed together). In our prior publications, we developed computational pipelines that integrated bioinformatics and visual analytics software to accomplish research objectives [14,15,16]. We have also identified a possible stress response–equipped biological process involving genes for sirtuin and the universal stress protein in *Bacillus megaterium* [16].

A *Lactobacillus plantarum* gene for the universal stress protein is involved in response to phenolic acid compounds found in food products of plant origin [17,18]. The availability of over 500 genome sequences of *Lactobacillus* species presents opportunities to advance research on *Lactobacillus* universal stress proteins. The first objective of the research study reported in this article was to determine the distribution of the protein sequence length and protein domain architecture of lactobacilli universal stress proteins. This objective enabled us to collect comprehensive functional annotation information of lactobacilli universal stress proteins by integrating data sets on genome annotation and protein annotation for 593 lactobacilli genomes.

The protein domain architecture is the sequential order of domains in a protein sequence [19,20]. The protein length could provide insights into the presence of multiple protein domains [21]. The universal stress protein domain consists of 140 to 160 amino acids (aa) [22,23]. Therefore, the second objective of the research investigation was to evaluate the transcription direction and functions of genes adjacent to genes encoding universal stress proteins in genomes of lactobacilli species that were previously investigated for human body weight modification. Through both objectives we expected to accomplish the goal of identifying biological processes in lactobacilli that could influence the mechanisms for the extraction of energy from the diet in the human gastrointestinal system.

Our research strategies led to the identification of operon gene arrangements that include a gene for the universal stress protein as well as genes for dipeptide breakdown, manganese ion transport or mitochondrial fatty acid oxidation. In particular, the adjacent gene (*lp_1700*) of the *Lactobacillus plantarum* USP gene (*lp_1701*) has the annotation of sensory protein (pfam03073 (TspO_MBR)). This protein is also referred to as the Translocator Protein 18 kDa (TSPO) and found in prokaryotes and eukaryotes [24]. Recent evidence indicates TSPO has a stress response function and can modulate mitochondrial fatty acid oxidation [25,26]. We also found that the modulation of mitochondrial fatty acid oxidation is a demonstrated function of TSPO and an anti-obesity intervention. Our evaluation of the gene neighborhood of the only gene for the universal stress protein family (Amc_0071) in the probiotic *Akkermansia muciniphila* genome revealed an operonic arrangement with the gene for tryptophanyl-transfer ribonucleic acid (tRNA) synthase, in which the human homology is associated with the waist-hip ratio (WHR) and body fat distribution. These findings present new directions for research and public health education on obesity.

## 2. Methods

The methods employed in the reported research investigation consisted of a combination of methods from bioinformatics and visual analytics disciplines. The process can be broadly divided into the construction of data sets and the development of interactive visual representations of the data sets. Data sets included (i) functional annotations of lactobacilli genes that encode the USP domain (pfam00582); (ii) genome statistics and metadata annotation of lactobacilli genomes; and (iii) function and transcription direction of adjacent genes of lactobacilli USP genes.

### 2.1. Collection of Data on Lactobacilli Universal Stress Proteins and Lactobacilli Genomes

Genes encoding the USP domain in lactobacilli genomes were retrieved from the Integrated Microbial Genomes and Microbiomes (IMG/M) System using the Function Search Tool [27] (https://img.jgi.doe.gov/). The search term was pfam00582 and restricted to the Pfam list. The IMG Gene Cart tool was then used to select data from the annotation fields for Gene, Scaffold/Contig, Function and Project Metadata. In the case of the lactobacilli genomes, the annotations for Genome Field, Metadata and Data Statistics for lactobacilli genomes were retrieved from the IMG System’s genome browser tool. The data tables were exported to a spreadsheet file for subsequent analyses.

### 2.2. Visual Analytics of Information on Genome Annotation and Protein Annotation

Since the research investigation had a goal of understanding from diverse information, we used visual analytics strategy to support the performance of human-information interaction. The three spreadsheet files (genes, genomes and protein domain architecture) were imported into Tableau Software Desktop Professional version (Tableau Software, Seattle, WA, USA). Interactive visual representations were constructed to accomplish the objective: to determine protein length distribution and protein domain architecture of lactobacilli universal stress proteins.

### 2.3. Evaluation of the Genomic Context of Genes Encoding Universal Stress Proteins

The genomic context of genes encoding universal stress proteins were obtained through interaction with information on the respective gene pages in BioCyc Pathway/Genome Database Collection [28] and the IMG/M. The following features were used in the evaluation process (1) BioCyc (genome overview, transcription unit annotation and the alignment constructed with the multi genome browser); (2) IMG/M (chromosomal cassette search and chromosomal cassette alignment). The presence of a USP gene in a transcription unit as well as the strand location of genes (transcription direction) were evaluated to understand the molecular mechanisms of lactobacilli that influence carbohydrate and lipid metabolism, immune system and endocrine functions in humans.

The presence of a gene in a transcription unit was computationally extracted from text in the genome overview pages of lactobacilli genomes in the BioCyc collection (as of 5 May 2016 version 20.0). For example the genome overview page of *Lactobacillus plantarum* WCFS1 is located at [29]. To facilitate decision making on further evaluation on genes, genes that are part of operons were scored a “1”, otherwise “0”. Thus a data set of operon status of genes in lactobacilli genomes in the BioCyc Collection was constructed. The transcription unit annotation was viewed from the BioCyc gene page. For example, the gene page for the universal stress protein with locus tag *lp_2993* in *L. plantarum* WCFS1 genome is located at [30]. The corresponding gene page for *lp_2993* in the IMG/M can be obtained with Gene ID 637389959.

A three-digit transcription direction code was constructed for selected lactobacilli USP genes to document the transcription direction of the adjacent genes relative to the gene encoding the universal stress protein domain. Thus a data set that includes locus tags and the three-digit binary transcription direction code was constructed to represent the strand location (+ or −) of the USP genes and adjacent genes. The middle digit of the binary code was always assigned to 1. If the adjacent gene is on same strand a “1”, otherwise a “0” was assigned. Therefore, the following binary codes were possible: 010, 110, 011, and 111. The binary transcription direction codes were constructed from genome annotation files (RefSeq) obtained from the PathoSystems Resource Integration Center’s File Transfer Protocol (FTP) Server located at [31].

The data set construction process requires the computational processing of multiple files. Thus we used the Blue Waters Supercomputer (NCSA, University of Illinois, Urbana, IL, USA) [32], to construct the data sets obtained from the bioinformatics resources. The combination of the three-digit binary transcription direction code and the one-digit binary operon status code enabled us to identify biological processes that involved universal stress proteins and other proteins. Visual analytics of the genomic context data sets and protein functional annotation were conducted in Tableau Software (Desktop Professional version).

## 3. Results

A total of 2788 genes annotated to encode the universal stress protein domain (pfam00582) in 593 lactobacilli genomes (40 Draft, 88 Finished and 465 Permanent Draft) were obtained from the Integrated Microbial Genomes and Microbiomes (IMG/M version 4.560, March 2016). The results for the components of the research investigation are presented in the section below. Interactive visual representations are available at [33].

### 3.1. Protein Length and Protein Domain Architecture

**Figure 1:**
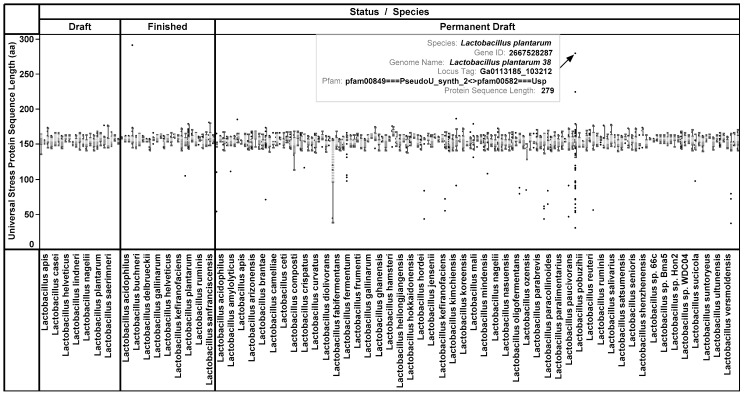
An overview of the protein sequence lengths of universal stress proteins of *Lactobacillus* species. Majority (2714 of 2788) of the protein sequences are 131 aa to 200 aa. Protein sequences greater than 200 aa in length could be multi-domain proteins. For example Ga0113185_103212 in *L. plantarum* 38 has a protein length of 291 aa and is composed of two domains: RNA pseudouridylate synthase (PseudoU_synth_2) and universal stress proteins (USP).

Among the 2788 USPs, 93 length types were observed and the lengths ranged from 30 aa to 896 aa. Based on the length of the domain (140 aa to 160 aa), we grouped sequences into three categories (i) 30 aa to 130 aa (58 sequences); (ii) 131 aa to 200 aa (2714 sequences); and 200 aa to 896 aa (six sequences). An overview box plot is presented in Figure 1.

Five of the 2788 universal stress protein sequences were annotated with at least one other protein domain. There was one USP each from *L. ghanensis* DSM 18630, *L. paracasei paracasei* CNCM I-4270, and *L. satsumensis* DSM 16230 that had the following additional domains: pfam13493 (DUF4118); pfam02518 (HATPase_c); pfam02702 (KdpD); pfam00512 (HisKA). In *Lactobacillus plantarum* 38, additional domains co-located with the USPs were pfam00849 (RNA pseudouridylate synthase (PseudoU_synth_2)) and pfam10400 (Virulence activator alpha C-term (Vir_act_alpha_C)).

**Figure 2:**
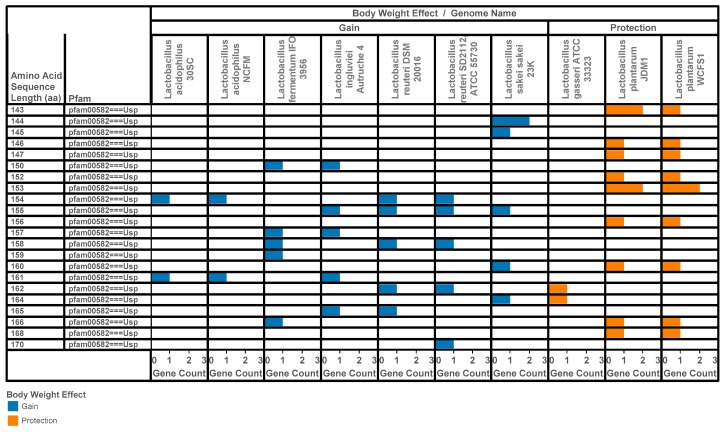
Visual representation integrating information on patterns of protein length, protein domain and count of genes for universal stress protein per lactobacilli genome. The 10 genomes are representative of lactobacilli species investigated for a metabolic role in modifying body weight [8].

Figure 2 presents patterns of protein length, protein domain and count of genes for the universal stress protein per genome of 10 representative *Lactobacillus* species investigated for a metabolic role in modifying body weight. We observed 53 USP genes annotated in the 10 lactobacilli genomes. The gene counts per genome are two (*Lactobacillus acidophilus*, *Lactobacillus gasseri*); five (*Lactobacillus fermentum*, *Lactobacillus reuteri*); six (*Lactobacillus sakei sakei*); 10 or 11 (*Lactobacillus plantarum*). There were 22 protein length types within the range of 143 aa to 170 aa. Within this length range, the following protein sequence lengths (aa) were not observed: 148, 149, 151, 163 and 167. Three genomes (*L. sakei sakei* 23K, *L. plantarum* JDM1 and *L. plantarum* WCFS1) had instances where two USPs had identical lengths.

### 3.2. Identification of Lactobacilli Universal Stress Proteins Encoded in Operons

We analyzed the gene neighborhood of 43 genes encoding universal stress proteins predicted from the genomes of eight representative *Lactobacillus* species investigated for body weight modification. A data set was constructed that included gene identifiers (BioCyc, IMG/M, RefSeq Locus Tag); operon status (0 or 1); transcription direction code (010, 011, 110, 111); gene product description for adjacent genes; position of adjacent gene (before or after); and lactobacilli weight modification annotation (protection or gain).

The predicted protein products (with pfam protein domain functions) encoded in the same operon as a gene for a lactobacilli universal stress protein (Table 1) were 2-dehydropantoate 2-reductase (pfam02558 (ApbA), pfam08546 (ApbA_C)); dipeptidase PepV (pfam01546 (Peptidase_M20)); manganese transport protein (pfam01566 (Nramp)); and sensory protein (pfam03073 (TspO_MBR)). Additionally, predicted protein products (with Pfam protein domain functions) in the same transcription direction with an operonic lactobacilli USP gene included (1) elongation factor G (pfam00009 (GTP_EFTU)); and (2) N-acetylmuramoyl-L-alanine amidase (pfam01832 (Glucosaminidase).

### 3.3. Alignment of Genomic Regions for Two-Gene Operons that include Gene for Universal Stress Protein

A total of nine universal stress proteins (*Lactobacillus sakei*: LSA0042, LSA0247; *Lactobacillus reuteri*: LAR_1223; *Lactobacillus plantarum*: lp_1322 (JDM1_1117), lp_1701 (JDM1_1432) and lp_2993 (JDM1_2397)) in the weight-modifying lactobacilli were identified as part of two-gene operons. We used the multi-genome browser tool at BioCyc and the chromosomal cassette tool in IMG/M to generate visualizations of relatedness of the genomic context of the two-gene operons among the lactobacilli genomes investigated. The operon encoding protein domains pfam00582 (Usp) and pfam03073 (TspO_MBR) was unique to *Lactobacillus plantarum* genomes.

**Figure 3:**
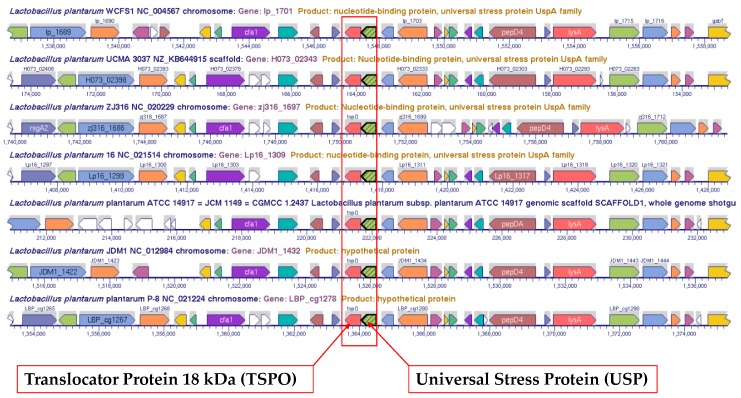
Multi-genome alignment of the genomic region in *Lactobacillus plantarum* genomes that includes the two-gene operon encoding translocator protein 18 KDa and universal stress protein.

Chromosomal cassette alignment, chromosomal cassette search and multi-genome alignment confirmed the conservation of the two-gene operon in *Lactobacillus plantarum* (Figure 3). In a collection of 3546 completely sequenced “Finished” bacteria genomes, only the 13 *Lactobacillus plantarum* genomes had genes for Usp and TspO_MBR domains as neighbors. Using the genome of *L. plantarum* WCFS1 as the reference, the genes tagged as *lp_1702* and *lp_1703* and respectively annotated as membrane protein and diacylglycerol kinase family protein were in the same transcription direction as the two-gene operon.

### 3.4. Gene Neighborhood of Gene for the Universal Stress Protein in *Akkermansia muciniphila* Genome

**Figure 4:**
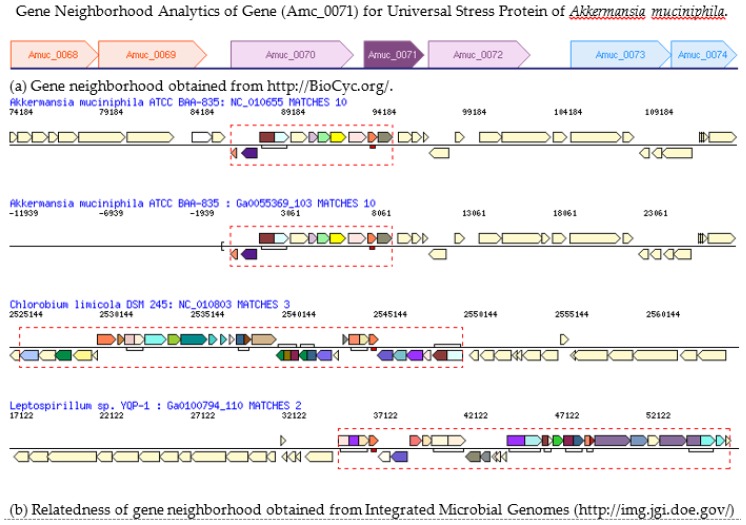
Gene neighborhood analytics of gene (*Amc_0071*) for universal stress protein of *Akkermansia muciniphila* ATCC BAA-835. (**a**) Gene neighborhood of gene for universal stress protein (*Amc_0071*) in *Akkermansia muciniphila*; (**b**) Relatedness of gene neighborhood of gene for universal stress protein (gene with red rectangle) in *Akkermansia muciniphila* to other genomes in the Integrated Microbial Genomes system. Two genome sequences of *Akkermansia muciniphila* ATCC BAA-835 are available in the IMG/M system. Each genome encodes one gene for universal stress protein [37].

The presence of a two-gene operon annotated for energy metabolism and stress response functions in *Lactobacillus plantarum* encouraged us to investigate the gene neighborhood of the only gene for universal stress protein encoded in the genome of *Akkermansia muciniphila* ATCC BAA-835. The abundance of *Akkermansia muciniphila* declines with the onset of high-fat diet-induced obesity in rodents [34]. Furthermore, the abundance of *Akkermansia muciniphila* is associated with the healthiest metabolic status in overweight/obese humans [1]. Gene neighborhood analysis (with BioCyc and IMG/M) revealed that adjacent genes of Amuc_0071, the USP gene for *Akkermansia muciniphila* ATCC BAA-835, are *Amuc_0070* (WARS), trpS tryptophanyl-tRNA synthetase (EC:6.1.1.2) and *Amuc_0072* (metallophosphoesterase). The three-gene loci of *Amuc_0070*, *Amuc_0071*, and *Amuc_0072* are predicted to be in same operon (Figure 4a). The alignment of the genomic region containing the three-gene operon confirmed the uniqueness of the adjacency of the *Akkermansia muciniphila* USP gene (Figure 4b). A search of literature databases for tryptophanyl-tRNA synthase and obesity identified reports of association with the waist-hip ratio and body fat distribution [35,36].

## 4. Discussion

In this present study, we have collected comprehensive functional annotation information on 2788 lactobacilli universal stress proteins by integrating data sets on genome annotation and protein annotation for 593 lactobacilli genomes. The determinants that could influence obesity development are the USPs and their operon partners as well as non-operonic gene neighbors associated with the carbohydrate or lipid metabolism. Several *Lactobacillus* strains have been grouped as weight protection or weight gain species [8]. The developed mechanisms observed for weight protection–associated *Lactobacillus* species are defense against enhanced glycolysis and defense against oxidative stress. However, the limited ability to break down fructose or glucose was due to mechanisms observed in genomes of weight gain–associated *Lactobacillus* species. Our focus on the genomic regions encoding the universal stress proteins has revealed genomic evidence for bacterial mechanisms that could influence obesity development.

The availability of genomic information on probiotic bacteria with proteins to modify human body weight presents opportunities to identify novel bacteria determinants of obesity development [7,8]. Several strains of *Lactobacillus plantarum* have been identified in multiple studies as having anti-obesity effects [7,8,38,39,40,41]. Data from our research study revealed a *L. plantarum* gene for the universal stress protein that is in operon association with a gene for fatty acid oxidation. This is the first report describing the conservation in *Lactobacillus plantarum* of an operon encoding the universal stress protein and Translocator Protein 18 kDa (TSPO).

The TSPO is a membrane protein found in prokaryotes and eukaryotes [24]. The bacterial TSPO can function as a substitute for the eukaryotic ortholog [25,42]. There are diverse functions proposed or demonstrated for TSPO in mammals including cholesterol transport, sex steroid biosynthesis, axonal regeneration and neuroinflammation [24,25,43]. Recent studies demonstrated that TSPO has a stress response function and can modulate mitochondrial fatty acid oxidation [25,26]. Interestingly, fatty acid oxidation as a means of enhancing cellular energy expenditure is a proposed alternative anti-obesity intervention to reducing food intake and increasing physical exercise [44]. TSPO expression in adipose tissues is reduced in obesity as well as in the placenta of obese women [45]. A future research direction is to determine the extent to which the *Lactobacillus plantarum* TSPO (*Lp*TSPO) is able to oxidize dietary fatty acids.

Our gene neighborhood analytics using a combination of tools from the BioCyc and IMG/M bioinformatics resources revealed that adjacent genes of *Amuc_0071* (the USP gene for *Akkermansia muciniphila* ATCC BAA-835) are *Amuc_0070* (WARS, trpS tryptophanyl-tRNA synthetase (EC:6.1.1.2)) and *Amuc_0072* (metallophosphoesterase) (Figure 4). The essential tryptophanyl-tRNA synthetase ensures the translation of the genetic code for tryptophan through the activation of tryptophan by ATP and transfer to tRNATrp [46]. In humans, the tryptophanyl-tRNA synthetase exists as cytoplasmic (WARS) and mitochondrial (WARS2) with the mitochondrial form in genomic loci that are associated with the waist-hip ratio and fat distribution [35,36]. Interestingly, in a study of the abundance of *A. muciniphila* in overweight and obese adults before and after caloric restriction, *A. muciniphila* abundance was inversely related to the waist-hip ratio [1]. In the human genome, the genes for tryptophanyl-tRNA synthetase (WARS) and TBX15 (a transcription factor from the T-box family for adipocyte development) are neighbors [35]. Interestingly, the predicted gene neighborhood of *lp_0434* (tryptophanyl-tRNA synthetase) in *Lactobacillus plantarum* WCFS1 indicates an adjacent T-box RNA gene (*lp_R0092*, RF00230). Further research investigations are required to define the effects of *lp_0434* and lp_R0092 in the weight protection role of *L. plantarum*.

## 5. Conclusions

There are two major findings from this research study. The first finding is the *Lactobacillus plantarum* two-gene operon, consisting of the universal stress protein and the membrane translocator protein known to function in fatty acid oxidation. The second finding is the presence of a three-gene operon in weight-loss–associated *Akkermansia muciniphila* which includes a gene whose human mitochondrial homolog is associated with the waist-hip ratio and fat distribution. From a public health perspective, elucidation of the bacterial determinants influencing obesity will help in educating the public on optimal probiotic use for anti-obesity effects.

## Figures and Tables

**Table 1:** Predicted protein functions of neighboring genes of genes for universal stress protein in representative genomes of lactobacilli investigated for body weight–modifying characteristics.

Locus Tag	Protein Length (aa)	Genome Name	Operon Neighbor Gene Product
LSA0042	155	*Lactobacillus sakei sakei* 23K	2-dehydropantoate 2-reductase
LAR_1223	170	*Lactobacillus reuteri* JCM 1112	dipeptidase PepV
lp_1322	168	*Lactobacillus plantarum* WCFS1	dipeptidase PepV
JDM1_1117	168	*Lactobacillus plantarum* JDM1	dipeptidase PepV
lp_2993	143	*Lactobacillus plantarum* WCFS1	manganese transport protein
JDM1_2397	143	*Lactobacillus plantarum* JDM1	manganese transport protein
LSA0247	144	*Lactobacillus sakei sakei* 23K	manganese transport protein
lp_1701	152	*Lactobacillus plantarum* WCFS1	sensory protein
JDM1_1432	152	*Lactobacillus plantarum* JDM1	sensory protein

Protein Pfam families encoded in two-gene operon that includes a gene for universal stress protein. LSA0042: pfam02558 (ApbA) and pfam08546 (ApbA_C). LAR_1223, lp_1322, JDM1_1117: pfam01546 (Peptidase_M20). lp_2993, JDM1_2397, LSA0247: pfam01566 (Nramp). lp_1701, JDM1_1432: pfam03073 (TspO_MBR).
